# Evaluation of Project P.A.T.H.S. (Secondary 2 Program) by the Program Implementers: Findings Based on the Experimental Implementation Phase

**DOI:** 10.1100/tsw.2008.84

**Published:** 2008-05-23

**Authors:** Daniel T. L. Shek, Rachel C. F. Sun, Daniel W. M. Lung

**Affiliations:** ^1^ Department of Sociology, East China Normal University, Shanghai, China; ^2^ Kiang Wu Nursing College of Macau, Macau; ^3^ Social Welfare Practice and Research Centre, The Chinese University of Hong Kong, Hong Kong

**Keywords:** adolescence, education, youth development, human development, school, Chinese, Hong Kong

## Abstract

A total of 49 schools (N = 8,167 students) participated in the Secondary 2 Program of the Experimental Implementation Phase of the Project P.A.T.H.S. (Positive Adolescent Training through Holistic Social Programmes). After completion of the Tier 1 Program, 270 instructors completed the Subjective Outcome Evaluation Form (Form B) to assess their views of the program, their own performance, and perceived effectiveness of the program. Based on the consolidated reports submitted by the schools to the funding body, the research team aggregated the consolidated data to form a “reconstructed” overall profile on the perceptions of the program implementers. Results showed that high proportions of the instructors had positive perceptions of the program and their own performance, and over 90% of the instructors regarded the program as helpful to the program participants. These findings are consistent with the subjective outcome evaluation findings based on the perspective of the program participants.

## INTRODUCTION

The Project P.A.T.H.S. (**P**ositive **A**dolescent **T**raining through **H**olistic **S**ocial Programmes) is a two-tier, positive youth development program financially supported by the Hong Kong Jockey Club Charities Trust[1]. In the Tier 1 Program of the project, students in Secondary 1 to 3 normally receive 20 h of training at each grade, involving 40 teaching units that are developed with reference to 15 positive youth development constructs[2,3]. Prior to the Full Implementation Phase (when P.A.T.H.S. was launched in full scale), the Experimental Implementation Phase served the purpose of accumulating experience in program implementation and familiarizing front-line workers with the program design and philosophy. There were 52 secondary schools that joined the Secondary 1 Program of the Experimental Implementation Phase in the 2005/06 school year, and 49 out of these 52 secondary schools continued with the Secondary 2 Program of the Experimental Implementation Phase in the 2006/07 school year.

Based on the principle of triangulation, evaluation data based on different strategies and sources have shown the effectiveness of the Secondary 1 Program. These include objective outcome evaluation[4,5], subjective outcome evaluation based on quantitative and qualitative data collected from the program participants and instructors[6,7,8,9,10,11], process evaluation involving systematic observations of the delivery of program[12,13], interim evaluation[14,15], and qualitative evaluation, including focus groups based on students and program implementers[16,17], students' diaries[18], and case studies[19,20].

Although the above studies can give some support for the positive aspects of the program, it is worthwhile to examine whether similar positive outcomes would be obtained for the program when extended to the Secondary 2 level[21,22]. In the subjective outcome evaluation of the Secondary 2 Program, findings based on the perspective of the students showed that the program was helpful to them[23]. However, "because personnel, consciously or unconsciously, influence the effectiveness of prevention program lessons, it is important to assess their perceptions when evaluating a specific program to provide insight into the context in which the program operates" ([24]; p. 219); thus, it is important to understand the evaluation of the program based on the perspective of the program implementers.

In view of the fact that there was a paucity of research studies documenting the perceptions of workers[25], Shek et al.[7] put forward several arguments on the importance of examining the views of the program implementers in the evaluation of youth development programs. First, according to utilization-focused evaluation[26], in order "to achieve more reliable and valid evaluations, a number of data sources and perspectives should be combined" ([27]; p. 1225), and thus the program implementers are some of the stakeholders who should be involved in the evaluation process. Second, with reference to the principle of triangulation, the evaluation data based on various sources can help to cross-check the program effectiveness across the data collected from different sources and help to paint a full picture of program effects. Third, because the program implementers have professional training and experience, they may give a good assessment of program effectiveness. Fourth, the inclusion of program implementers' views when judging the program and their own performance can give them a sense of respect and fairness, and avoid biases that generated from the evaluation data based on the clients only. Finally, subjective outcome evaluation that includes questions that ask the program implementers about program implementation and their own performance can facilitate their reflective practice, which enhances professional growth and development[28,29].

Another limitation of the literature is that the relationships among different domains of subjective outcome evaluation are not clear. In a typical positive youth development program, subjective outcome evaluation can be directed to several areas, including one's perception of the program, program implementers, and effectiveness of the program. Are perceived characteristics of the program and program implementers related to perceived benefits of the program? Although it is intuitively appealing to hypothesize that perceived characteristics of the program and program implementers are positively related to perceived benefits of the program, empirical evidence regarding these hypotheses is not clear. As program implementer qualities (e.g., encouraging attitudes) might influence program characteristics (e.g., involvement of students), it was expected that perceived program characteristics would be positively related to program implementer characteristics (Hypothesis 1). Furthermore, as program outcomes depend on program input, it was predicted that program and program implementer characteristics would be positively related to perceived benefits of the program (Hypotheses 2 and 3).

In the present study, the subjective outcome evaluation findings of the Secondary 2 Program (Experimental Implementation Phase) based on the perspective of the program implementers are reported. As each participating school was required to submit an evaluation report with the consolidated subjective outcome evaluation profile of the school to the funding body, the research team made use of such reports to "reconstruct" the overall profile of the subjective outcome evaluation data. The major advantage of this strategy is that we can practice evaluation in the field while, at the same time, conduct secondary data analyses of the reports submitted. Besides looking at the overall profile, the relationships between perceived program and implementer characteristics, and perceived benefits of the program would be examined.

## METHODS

### Participants and Procedures

There were 49 schools that joined the Secondary 2 Program of the Experimental Implementation Phase of Project P.A.T.H.S. A total of 336 instructors (including social workers, teachers, and teaching assistants) implemented the program in the schools. The mean numbers of social workers and teachers implementing the program per school were 2.27 (range: 0–6) and 4.55 (range: 0–12), respectively. The mean number of students per school was 166.67 (range: 32–240 students), with an average of 4.51 classes per school (range: 1–7 classes). Among the participating schools, 22 adopted the full program (i.e., 20-h program involving 40 units), while the other 27 schools adopted the core program (i.e., 10-h program involving 20 units). The mean number of sessions used to implement the program was 23.76 (range: 10–40 sessions). While 26 (53.06%) schools incorporated the program in the formal curriculum (e.g., Liberal Studies, Life Education), 23 schools (46.94%) used other modes (e.g., form master's periods and other combinations) to implement the program. The average level of program adherence reported by the instructors was 84.27%, with a range from 50 to 100%.

After the Tier 1 Program was completed, a total of 270 instructors responded to the Subjective Outcome Evaluation Form (Form B) developed by the research team in a self-administration format and anonymous manner. The data collection was normally carried out after the completion of the program. To facilitate the program evaluation, the research team developed an evaluation manual with standardized instructions for collecting the subjective outcome evaluation data[30]. In addition, adequate training was provided to the instructors during 20-h training workshops on how to collect and analyze the data collected by Form B.

### Instruments

The Subjective Outcome Evaluation Form (Form B) was designed by Shek and Siu[30]. Broadly speaking, there are several parts in this evaluation form as follows:

- Program implementers' perceptions of the program, such as program objectives, design, classroom atmosphere, interaction among the students, and the students' participation during class (10 items).
- Program implementers' perceptions of their own practice, including their understanding of the course, teaching skills, professional attitude, involvement, and interaction with the students (10 items).
- Program implementers' perceptions of the effectiveness of the program, such as promotion of different psychosocial competencies, resilience, and overall personal development of the students (16 items).
- The extent to which the program implementers would recommend the program to other students with similar needs (1 item).
- The extent to which the program implementers would teach similar programs in the future (1 item).
- The extent to which the program enhanced the program implementers' professional growth (1 item).
- Things that the program implementers obtained from the program (open-ended question).
- Things that the program implementers appreciated most (open-ended question).
- Difficulties encountered (open-ended question).
- Areas that require improvement (open-ended question).

For the closed-ended questions, the workers who collected the data were requested to input the data into an EXCEL file developed by the research team that would automatically compute the frequencies and percentages associated with the different ratings for an item. The schools were required to submit the evaluation reports, together with the soft copy of the consolidated data sheets, to the funding body. After receiving the consolidated data from the funding body, the research team aggregated the data to "reconstruct" the overall profile based on the subjective outcome evaluation data in an anonymous manner.

## RESULTS

Reliability analysis with the schools as the unit of analyses showed that Form B was internally consistent: 10 items related to the program (alpha = 0.96, mean interitem correlation = 0.71), 10 items related to the instructor (alpha = 0.88, mean interitem correlation = 0.44), 16 items related to the effectiveness (alpha = 0.97, mean interitem correlation = 0.70), and 39 items based on the whole Form B (alpha = 0.98, mean interitem correlation = 0.54).

This study presents the quantitative findings based on the closed-ended questions, from which several observations can be highlighted. First, about four-fifths of the instructors generally had positive perceptions of the program (Table 1), including clear curriculum objectives (92.94%), pleasant classroom atmosphere (85.50%), and well-planned activities (84.44%). Second, a high proportion of the instructors had a positive evaluation of their own performance (Table 2). For example, 99.11% of the instructors were ready to offer help to the students in need, 98.79% expressed that they were concerned about the students, and 96.92% believed that they had very good professional attitude. Third, as shown in Table 3, about four-fifths of the instructors perceived that the program promoted the development of students, including their bonding (89.93%), resilience (86.09%), social competence (92.16%), emotional competence (91.42%), moral competence (88.06%), self-awareness (92.54%), and overall development (92.13%). Fourth, 88.43% of the instructors would recommend the program to students with similar needs. Fifth, 82.71% of the instructors expressed that they would teach similar courses again in the future. Finally, over 85% of the instructors indicated that implementing the program helped their professional growth (Table 4).

Consistent with the proposed hypotheses, Table 5 shows that various domains of the subjective outcome evaluation were inter-related. First, results showed that the overall views about the program and the overall views about the instructors were significantly correlated with each other, thus providing support for Hypothesis 1. Second, consistent with Hypotheses 2 and 3, perceived program and program implementer characteristics were positively correlated with all 16 aspects of the perceived effectiveness of the program.

## DISCUSSION

This subjective outcome evaluation study evaluated the Tier 1 Program of the Secondary 2 Program of the Project P.A.T.H.S. based on the perspective of the program implementers. Several observations could be highlighted. First, a high proportion of the instructors had positive perceptions of the program, including the program design, their own performance, and program effectiveness in the second year of the Experimental Implementation Phase. Moreover, when compared with the results obtained from the first year of the Experimental Implementation Phase, there were increments in the percentage of positive responses in most of the items. Second, program adherence level reported by the program implementers was relatively high (mean = 84.27%, range = 50–100%), which is consistent with that reported by the observers in the process evaluation (mean = 83.6%, range = 70–95%)[22]. Research findings showed that program implementers with positive views about the program and high program adherence were two crucial factors for program effectiveness[31,32].

**TABLE 1**
**Views of the Program Implementers about the Program**

| | 1 | | 2 | | 3 | | 4 | | 5 | | 6 | | Participants with Positive Responses (Option 4–6) | |
|---|---|---|---|---|---|---|---|---|---|---|---|---|---|---|
| | Strongly Disagree | | Disagree | | Slightly Disagree | | Slightly Agree | | Agree | | Strongly Agree | | | |
| | N | % | N | % | N | % | N | % | N | % | N | % | N | % |
| The objectives of the curriculum are very clear. (N = 269) | 1 | 0.37 | 6 | 2.23 | 12 | 4.46 | 71 | 26.39 | 164 | 60.97 | 15 | 5.58 | 250 | 92.94 |
| The design of the curriculum is very good. (N = 267) | 4 | 1.50 | 21 | 7.87 | 33 | 12.36 | 100 | 37.45 | 104 | 38.95 | 5 | 1.87 | 209 | 78.28 |
| The activities were carefully planned. (N = 270) | 2 | 0.74 | 12 | 4.44 | 28 | 10.37 | 105 | 38.89 | 113 | 41.85 | 10 | 3.70 | 228 | 84.44 |
| The classroom atmosphere was very pleasant. (N = 269) | 1 | 0.37 | 12 | 4.46 | 26 | 9.67 | 122 | 45.35 | 98 | 36.43 | 10 | 3.72 | 230 | 85.50 |
| There was much peer interaction among the students. (N = 270) | 1 | 0.37 | 11 | 4.07 | 35 | 12.96 | 119 | 44.07 | 94 | 34.81 | 10 | 3.70 | 223 | 82.59 |
| Students participated actively during lessons (including discussions, sharing, games, etc.). (N = 270) | 3 | 1.11 | 11 | 4.07 | 38 | 14.07 | 119 | 44.07 | 87 | 32.22 | 12 | 4.44 | 218 | 80.74 |
| The program has a strong and sound theoretical support. (N = 268) | 0 | 0.00 | 15 | 5.60 | 32 | 11.94 | 96 | 35.82 | 107 | 39.93 | 18 | 6.72 | 221 | 82.46 |
| The teaching experience I encountered enhanced my interest in the course. (N = 269) | 3 | 1.12 | 20 | 7.43 | 42 | 15.61 | 104 | 38.66 | 94 | 34.94 | 6 | 2.23 | 204 | 75.84 |
| Overall speaking, I have very positive evaluation of the program. (N = 270) | 7 | 2.59 | 16 | 5.93 | 33 | 12.22 | 109 | 40.37 | 98 | 36.30 | 7 | 2.59 | 214 | 79.26 |
| On the whole, students like this curriculum very much. (N = 270) | 4 | 1.48 | 16 | 5.93 | 36 | 13.33 | 115 | 42.59 | 95 | 35.19 | 4 | 1.48 | 214 | 79.26 |

**TABLE 2**
**Views of the Program Implementers about Themselves**

| | 1 | | 2 | | 3 | | 4 | | 5 | | 6 | | Participants with Positive Responses (Option 4–6) | |
|---|---|---|---|---|---|---|---|---|---|---|---|---|---|---|
| | Strongly Disagree | | Disagree | | Slightly Disagree | | Slightly Agree | | Agree | | Strongly Agree | | | |
| | N | % | N | % | N | % | N | % | N | % | N | % | N | % |
| I have a good mastery of the curriculum. (N = 266) | 1 | 0.38 | 8 | 3.01 | 29 | 10.90 | 106 | 39.85 | 117 | 43.98 | 5 | 1.88 | 228 | 85.61 |
| I prepared well for the lessons. (N = 266) | 0 | 0.00 | 8 | 3.01 | 26 | 9.77 | 114 | 42.86 | 110 | 41.35 | 8 | 3.01 | 232 | 86.05 |
| My teaching skills were good. (N = 264) | 0 | 0.00 | 5 | 1.89 | 24 | 9.09 | 123 | 46.59 | 105 | 39.77 | 7 | 2.65 | 235 | 88.21 |
| I have good professional attitudes. (N = 266) | 0 | 0.00 | 0 | 0.00 | 11 | 4.14 | 72 | 27.07 | 168 | 63.16 | 15 | 5.64 | 255 | 96.92 |
| I was very involved. (N = 267) | 0 | 0.00 | 3 | 1.12 | 12 | 4.49 | 84 | 31.46 | 146 | 54.68 | 22 | 8.24 | 252 | 94.83 |
| I gained a lot during the course of instruction. (N = 267) | 4 | 1.50 | 6 | 2.25 | 32 | 11.99 | 115 | 43.07 | 99 | 37.08 | 11 | 4.12 | 225 | 83.23 |
| I cared for the students. (N = 267) | 0 | 0.00 | 0 | 0.00 | 6 | 2.25 | 51 | 19.10 | 166 | 62.17 | 44 | 16.48 | 261 | 98.79 |
| I was ready to offer help to students when needed. (N = 266) | 0 | 0.00 | 0 | 0.00 | 2 | 0.75 | 38 | 14.29 | 182 | 68.42 | 44 | 16.54 | 264 | 99.11 |
| I had much interaction with the students. (N = 267) | 0 | 0.00 | 5 | 1.87 | 17 | 6.37 | 109 | 40.82 | 128 | 47.94 | 8 | 3.00 | 245 | 91.98 |
| Overall speaking, I have a very positive evaluation of myself as an instructor. (N = 267) | 0 | 0.00 | 5 | 1.87 | 12 | 4.49 | 71 | 26.59 | 173 | 64.79 | 6 | 2.25 | 250 | 96.04 |

Third, over 90% of the instructors had positive perceptions about themselves, particularly on their professional attitude (items 4 and 5 in Table 2) and interaction with students (items 7, 8, and 9 in Table 2). The findings revealed that the instructors implemented the program in a devoted manner, and most of them were willing to build up rapport with the students and to offer help to the students in need. Fourth, the program was perceived by the program implementers as effective in a sense that it was not only beneficial to the development of the students, but also helpful to their own professional growth.

While the present findings can be interpreted in terms of positive program effects, four alternative explanations are present[7]. The first alternative explanation is that as the instructors are personally involved in the program implementation, they tend to judge the program effect and their own performance in a favorable manner according to the "beauty on the beholder side" hypothesis. The second alternative explanation is that as the instructors may buy-in the value of the program or have high professional self-esteem, they would find it difficult to rate the program and themselves in an unfavorable manner with

**TABLE 3**
**Perceived Effectiveness of the Program by the Program Implementers**

| The extent to which the Tier 1 Program (i.e., the program in which all students have joined) has helped your students. | 1 Unhelpful | | 2 Not Very Helpful | | 3 Slightly Helpful | | 4 Helpful | | 5 Very Helpful | | Participants with Positive Responses (Option 3–5) | |
|---|---|---|---|---|---|---|---|---|---|---|---|---|
| | N | % | N | % | N | % | N | % | N | % | N | % |
| It has strengthened students' bonding with teachers, classmates, and families. (N = 268) | 2 | 0.75 | 25 | 9.33 | 129 | 48.13 | 105 | 39.18 | 7 | 2.61 | 241 | 89.93 |
| It has strengthened students' resilience in adverse conditions. (N = 266) | 1 | 0.38 | 36 | 13.53 | 134 | 50.38 | 90 | 33.83 | 5 | 1.88 | 229 | 86.09 |
| It has enhanced students' social competence. (N = 268) | 0 | 0.00 | 21 | 7.84 | 103 | 38.43 | 132 | 49.25 | 12 | 4.48 | 247 | 92.16 |
| It has improved students' ability in handling and expressing emotions. (N = 268) | 1 | 0.37 | 22 | 8.21 | 128 | 47.76 | 109 | 40.67 | 8 | 2.99 | 245 | 91.42 |
| It has enhanced students' cognitive competence. (N = 267) | 1 | 0.37 | 39 | 14.61 | 120 | 44.94 | 99 | 37.08 | 8 | 3.00 | 227 | 85.02 |
| Students' ability to resist harmful influences has been improved. (N = 267) | 0 | 0.00 | 42 | 15.73 | 126 | 47.19 | 93 | 34.83 | 6 | 2.25 | 225 | 84.27 |
| It has strengthened students' ability to distinguish between the good and the bad. (N = 268) | 0 | 0.00 | 32 | 11.94 | 112 | 41.79 | 113 | 42.16 | 11 | 4.10 | 236 | 88.06 |
| It has increased students' competence in making sensible and wise choices. (N = 266) | 1 | 0.38 | 32 | 12.03 | 120 | 45.11 | 102 | 38.35 | 11 | 4.14 | 233 | 87.59 |
| It has helped students to have life reflections. (N = 267) | 2 | 0.75 | 43 | 16.10 | 99 | 37.08 | 103 | 38.58 | 20 | 7.49 | 222 | 83.15 |
| It has reinforced students' self-confidence. (N = 268) | 4 | 1.49 | 46 | 17.16 | 121 | 45.15 | 91 | 33.96 | 6 | 2.24 | 218 | 81.34 |
| It has increased students' self-awareness. (N = 268) | 1 | 0.37 | 19 | 7.09 | 107 | 39.93 | 130 | 48.51 | 11 | 4.10 | 248 | 92.54 |
| It has helped students to face the future with a positive attitude. (N = 267) | 3 | 1.12 | 38 | 14.23 | 127 | 47.57 | 92 | 34.46 | 7 | 2.62 | 226 | 84.64 |
| It has helped students to cultivate compassion and care about others. (N = 267) | 1 | 0.37 | 34 | 12.73 | 127 | 47.57 | 93 | 34.83 | 12 | 4.49 | 232 | 86.89 |
| It has encouraged students to care about the community. (N = 268) | 4 | 1.49 | 55 | 20.52 | 123 | 45.90 | 73 | 27.24 | 13 | 4.85 | 209 | 77.99 |
| It has promoted students' sense of responsibility in serving society. (N = 267) | 3 | 1.12 | 53 | 19.85 | 131 | 49.06 | 70 | 26.22 | 10 | 3.75 | 211 | 79.03 |
| It has enriched the overall development of the students. (N = 267) | 3 | 1.12 | 18 | 6.74 | 119 | 44.57 | 114 | 42.70 | 13 | 4.87 | 246 | 92.13 |

reference to the "cognitive dissonance" hypothesis. The third alternative explanation is that the instructors may consciously respond in a "nice" manner to help the researchers to illustrate positive program effect. However, this alternative explanation could be partially dismissed because the instructors responded to the questionnaires in an anonymous manner and negative ratings were recorded (e.g., whether the instructors perceived the teaching experience enhanced their interest in the course). Moreover, previous research findings based on the study have shown that subjective outcome evaluation findings actually converged with objective outcome evaluation findings[33,34]. Finally, the fourth alternative explanation is that the high proportion of positive responses observed were, in fact, random responses. Nevertheless,

**TABLE 4**
**Other Aspects of Subjective Outcome Evaluation based on the Views of the Workers**

If you have a student/client whose needs and conditions are similar to those of your students who have joined the program, will you suggest that he/she participate in this program? (N = 268)

| 1 | | 2 | | 3 | | 4 | | Participants with Positive Responses (Option 3–4) | |
|---|---|---|---|---|---|---|---|---|---|
| Definitely Will Not Suggest | | Will Not Suggest | | Will Suggest | | Definitely Will Suggest | | | |
| N | % | N | % | N | % | N | % | N | % |
| 2 | 0.75 | 29 | 10.82 | 210 | 78.36 | 27 | 10.07 | 237 | 88.43 |

If there is a chance, will you teach similar programs again in the future? (N = 266)

| 1 | | 2 | | 3 | | 4 | | Participants with Positive Responses (Option 3–4) | |
|---|---|---|---|---|---|---|---|---|---|
| Definitely Will Not Teach | | Will Not Teach | | Will Teach | | Definitely Will Teach | | | |
| N | % | N | % | N | % | N | % | N | % |
| 5 | 1.88 | 41 | 15.41 | 191 | 71.80 | 29 | 10.90 | 220 | 82.71 |

Do you think the implementation of the program has helped you in your professional growth (e.g. enhancement of your skills)? (N = 264)

| 1 | | 2 | | 3 | | 4 | | 5 | | Participants with Positive Responses (Option 3–5) | |
|---|---|---|---|---|---|---|---|---|---|---|---|
| Unhelpful | | Not Very Helpful | | Slightly Helpful | | Helpful | | Very Helpful | | | |
| N | % | N | % | N | % | N | % | N | % | N | % |
| 5 | 1.89 | 31 | 11.74 | 127 | 48.11 | 87 | 32.95 | 14 | 5.30 | 228 | 86.36 |

this alternative explanation can be dismissed because reliability analyses showed that the whole scale was internally consistent, and the psychometric properties of Form B were highly comparable across studies[11].

There are several strengths of this study. First, the subjective outcome evaluation findings are based on a large sample size (N = 270 instructors involving 49 schools). Such a big sample size substantially enhanced the generalizability of the research findings to other teacher and social worker populations. Second, different aspects of subjective outcome, including views of the program, instructors, and perceived effectiveness, were covered in the study. Third, the present findings showed that the instruments used in Form B were reliable in assessing several aspects of subjective outcomes. Fourth, the present study demonstrates the strategy of "reconstructing" the overall profile of the subjective outcomes based on the reports submitted by the participating schools. Finally, the present findings provided support for the three hypotheses concerning the inter-relationships among the different aspects of subjective outcome evaluation, which can be regarded as pioneering in the literature on positive youth development.

**TABLE 5**
**Intercorrelations among Domains in Subjective Outcome Evaluation**

| Variables/Items | Views about Program | Views about Instructors |
|---|---|---|
| Views about program | 1 | 0.79* |
| Views about instructors | 0.79* | 1 |
| Perceived effectiveness of the program: | | |
| It has strengthened students' bonding with teachers, classmates, and families. | 0.82* | 0.66* |
| It has strengthened students' resilience in adverse conditions. | 0.63* | 0.62* |
| It has enhanced students' social competence. | 0.74* | 0.61* |
| It has improved students' ability in handling and expressing emotions. | 0.69* | 0.69* |
| It has enhanced students' cognitive competence. | 0.58* | 0.59* |
| Students' ability to resist harmful influences has been improved. | 0.56* | 0.48* |
| It has strengthened students' ability to distinguish between the good and the bad. | 0.66* | 0.67* |
| It has increased students' competence in making sensible and wise choices. | 0.61* | 0.63* |
| It has helped students to have life reflections. | 0.66* | 0.72* |
| It has reinforced students' self-confidence. | 0.70* | 0.75* |
| It has increased students' self-awareness. | 0.71* | 0.73* |
| It has helped students to face the future with a positive attitude. | 0.65* | 0.71* |
| It has helped students to cultivate compassion and care about others. | 0.66* | 0.72* |
| It has encouraged students to care about the community. | 0.64* | 0.73* |
| It has promoted students' sense of responsibility in serving society. | 0.65* | 0.67* |
| It has enriched the overall development of the students. | 0.79* | 0.70* |

*Note*: A Bonferroni adjustment to the alpha level (33 pairs of correlations) was used.

*$p < 0.002$

Nevertheless, there are some limitations of the study. First, as the data were reconstructed from the reports submitted by the schools, the unit of analysis was schools rather than individual workers. Second, the restricted response format of the closed-ended questions would limit the respondents' expression of negative experiences, and the quantitative findings would hardly unravel the inner world of the respondents[35,36]. Hence, in order to illuminate the quantitative findings, further effort to examine such negative responses by looking at the qualitative findings is necessary. Third, because the instructors implementing the Secondary 2 Program may not be the same as those implementing the Secondary 1 Program, comparison with the data collected from the first year of the Experimental Implementation Phase may not be meaningful. Despite these limitations, the present findings based on the perspective of the program implementers constitute additions to the existing literature of program evaluation, and suggest that the Secondary 2 Program and its implementation were perceived in a positive manner by the program implementers in the Experimental Implementation Phase.

## ACKNOWLEDGMENTS

The preparation for this paper and the Project P.A.T.H.S. were financially supported by the Hong Kong Jockey Club Charities Trust.
